# Conjugates of methylated cyclodextrin derivatives and hydroxyethyl starch (HES): Synthesis, cytotoxicity and inclusion of anaesthetic actives

**DOI:** 10.3762/bjoc.10.325

**Published:** 2014-12-19

**Authors:** Lisa Markenstein, Antje Appelt-Menzel, Marco Metzger, Gerhard Wenz

**Affiliations:** 1 Organic Macromolecular Chemistry, Saarland University, Campus C4.2, 66123 Saarbrücken, Germany; 2 Department of Tissue Engineering and Regenerative Medicine, University Hospital Würzburg, Röntgenring 11, 97070 Würzburg, Germany

**Keywords:** anaesthetics, complexation, cyclodextrin, LCST, lower critical solution temperature, midazolam, occupancy, polymer, sevoflurane, solubility, starch

## Abstract

The mono-6-deoxy-6-azides of 2,6-di-*O*-methyl-β-cyclodextrin (DIMEB) and randomly methylated-β-cyclodextrin (RAMEB) were conjugated to propargylated hydroxyethyl starch (HES) by Cu^+^-catalysed [2 + 3] cycloaddition. The resulting water soluble polymers showed lower critical solution temperatures (LCST) at 52.5 °C (DIMEB-HES) and 84.5 °C (RAMEB-HES), respectively. LCST phase separations could be completely avoided by the introduction of a small amount of carboxylate groups at the HES backbone. The methylated CDs conjugated to the HES backbone exhibited significantly lower cytotoxicities than the corresponding monomeric CD derivatives. Since the binding potentials of these CD conjugates were very high, they are promising candidates for new oral dosage forms of anaesthetic actives.

## Introduction

Cyclodextrins (CDs), α(1→4) linked cyclic oligomers of anhydroglucose, are produced nowadays in industrial scale [1]. CDs are able to complex hydrophobic or amphiphilic guest molecules in aqueous phase [2]. β-CD, the seven membered ring forms inclusion compounds with derivatives of benzene, naphthalene, adamantane, and many other moieties of similar sizes [3]. Applications of β-CD as complexing agents are limited because of low aqueous solubilities of β-CD and its complexes. Furthermore, the toxic potential of β-CD is known for a long time. β-CD can cause haemolysis due to extraction of cholesterol from cell walls [4–5]. Also the parenteral application of high doses of β-CD can cause kidney diseases [6–7]. Consequently, pharmaceutical applications of β-CD are restricted to oral dosage forms, such as piroxicam β-CD [8–10].

Toxicity of β-CD can be minimized by derivatisation [11–12]. Both, hydroxypropyl-β-CD (HP-β-CD) and sulfobutyl-β-CD are less toxic than β-CD, but they are less defined due to a statistical substitution pattern [13]. HP-β-CD often shows a reduced binding potential compared to β-CD [2,14]. On the other hand, methylation of β-CD leads to excellent solubilities in water and high binding potentials, but causes even higher toxicity compared to native β-CD. Among the methyl derivatives of β-CD the heptakis-2,6-di-*O*-methyl derivative, abbreviated as DIMEB [15], showed very high binding potentials [16] and was already discussed by Szejli as a very promising candidate for parenteral drug delivery [17], but it was placed back because of its high toxicity [5,18–19]. Therefore our aim was to conjugate DIMEB for the first time to a polymeric backbone, which should hinder cellular uptake. CD polymers are known to have a much lower toxic potential compared to CD monomers [20].

Native β-CD was already conjugated by esterification [21], reductive amination [22–25], amide coupling [26–27] and [2 + 3] cycloadditions [28] to both biogenic and synthetic polymers. Conjugation of CDs to polysaccharides like chitosane [29], alginate [24] and dextrane [28] is advantageous for the design of drug delivery systems because of the low toxicities and biodegradabilities of those polymers. Among the various coupling reactions, the [2 + 3] cycloaddition of alkynes and azides, the so-called Huisgen reaction [30] and its Cu^+^-catalyzed version, called click reaction [31–32], is of special interest because this coupling proceeds rapidly and specific in aqueous media and tolerates many functional groups. Mono-6-azido-6-deoxy-β-CD was already coupled by the click reaction to propargylated dextrane by Nielsen et al. [28]. We intended to conjugate the corresponding methylated mono-azido-β-CD derivatives to propargylated hydroxyethyl starch (HES). Advantages of HES are its very high aqueous solubility paired with good biocompatibility and its very low allergenic potential [33]. HES is in use for a long time for many parenteral applications, such as plasma expanders [34]. Its rate of bio-degradation increases with decreasing the degree of substitution (MS) of the hydroxyethyl side groups. Nowadays, HES with a molecular weight of *M*_w_ = 130 kDa and MS <0.5 is preferred [35]. In the following, we describe the conjugation of azido-functionalized methylated β-CD derivatives to propargylated HES, the evaluation of the toxicity of these new polymers and first binding studies for the hydrophobic anaesthetic ingredients sevoflurane and midazolam. Sevoflurane, currently applied as an inhalation anaesthetic [36], could be solubilized in water to allow further use in oral or parenteral dosage forms as an analgesic drug. Also the uptake of midazolam could be improved by complexation in CD derivatives [37–38].

## Results and Discussion

Cyclodextrin polymers were synthesized by copper-catalyzed [2 + 3] cycloaddition of methylated derivatives of mono-6-azido-6-deoxy-β-CD (β-CD-N_3_) and propargylated hydroxyethyl starch (HES). Furthermore, a partially oxidized propargylated HES was employed as hydrophilic polymer backbone.

**Scheme 1:**
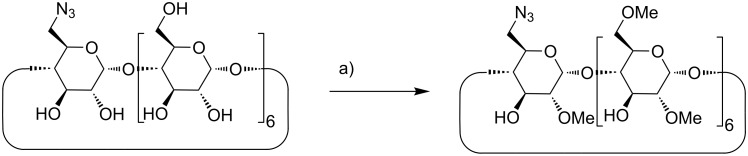
Synthesis of azido functionalized DIMEB **1a**. a) Ba(OH)_2_·8H_2_O/BaO/Me_2_SO_4_

The methylated β-CD-N_3_ was synthesized in a 3 step procedure starting from β-CD which was first converted via the 6-*O*-tosylate to β-CD-N_3_ following the procedures of Hocquelet et al. who also described the permethylated β-CD-N_3_ [39]. Furthermore, we headed for the regioselective 2,6-dimethylated product. Therefore β-CD-N_3_ was carefully methylated by dimethyl sulfate and a mixture of Ba(OH)_2_·8H_2_O and BaO as the base following the procedure of Szejtli et al. published for the methylation of native β-CD [15] (Scheme 1).

**Figure 1:**
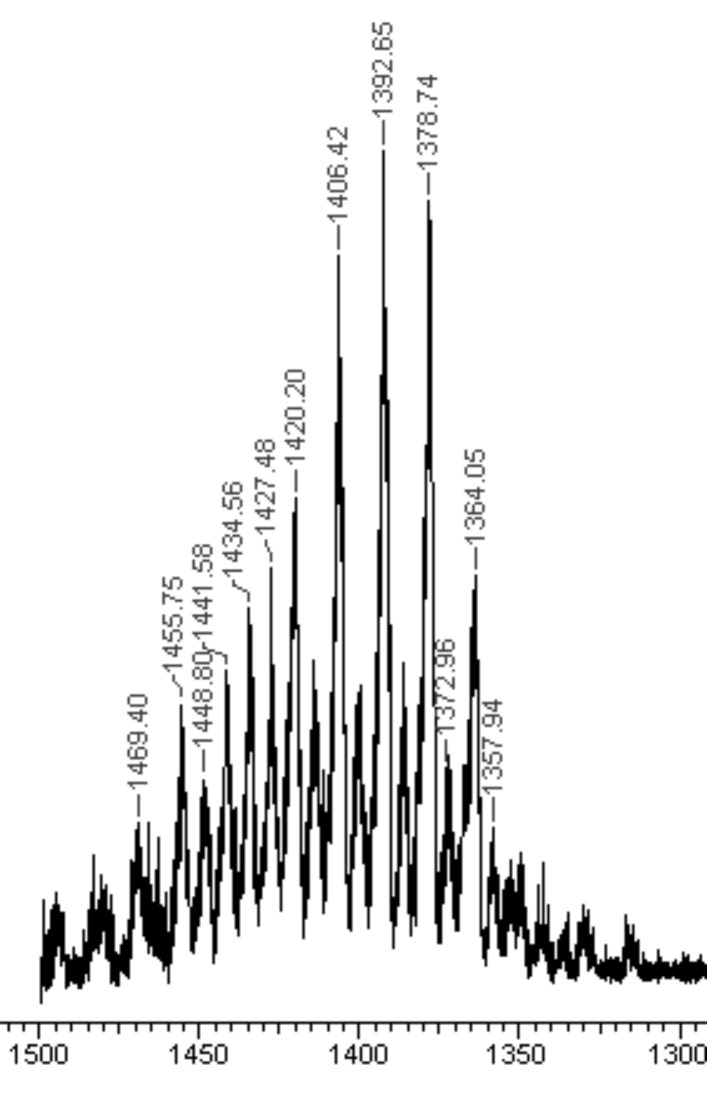
ESI mass spectrum of azido functionalized DIMEB **1a**.

The product **1a** showed a quite narrow distribution of methyl substituents as revealed by the ESIMS showing signals corresponding to β-CD-N_3_ derivatives with 14 to 18 methyl groups (Figure 1). From the average number of the molecular weight obtained from the ESIMS, *M*_n_ = 1383.6 g mol^−1^ a degree of substitution of methyl groups DS_CD_(methyl) = 2.2 per glucose unit was derived.

**Figure 2:**
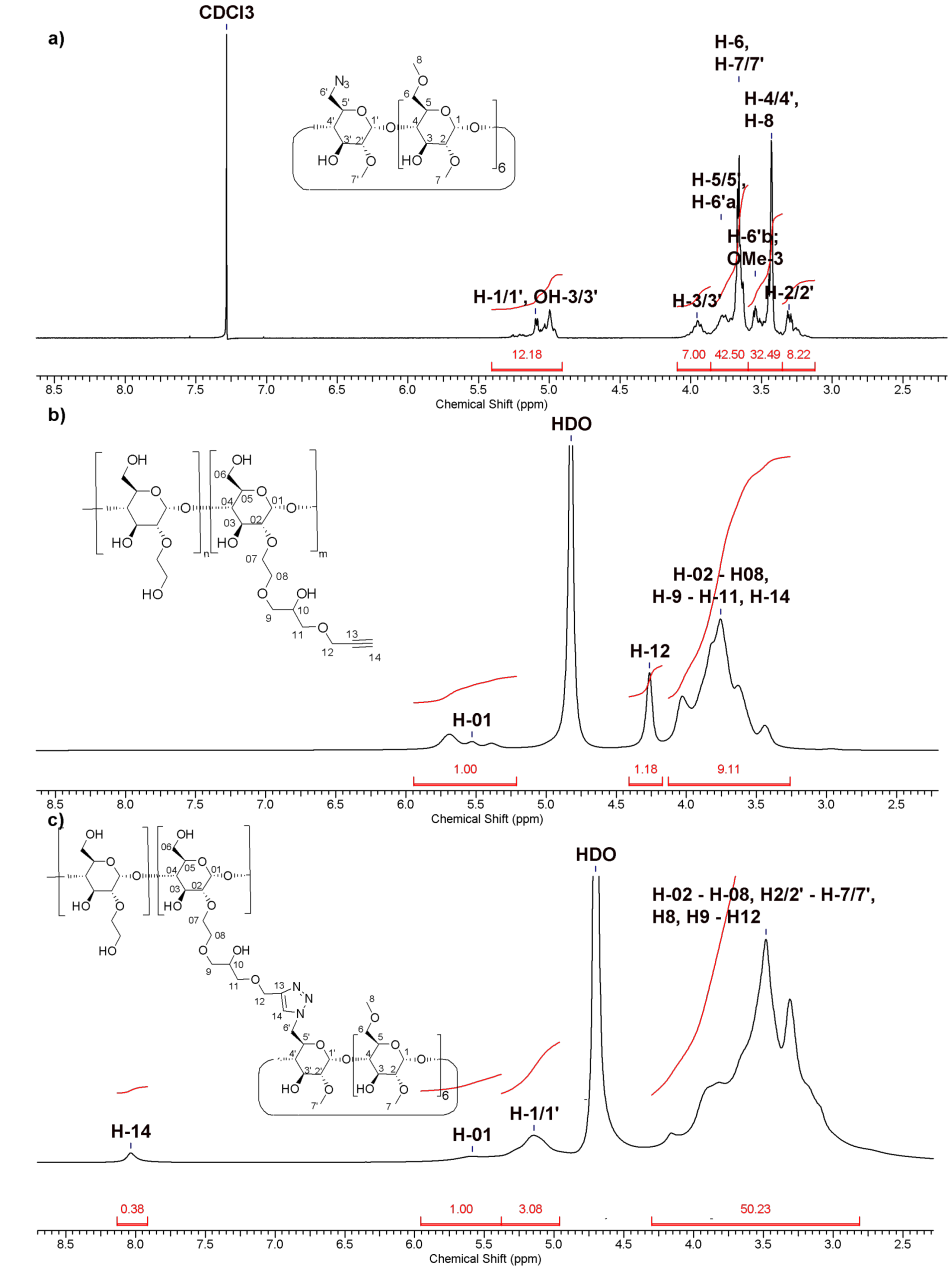
^1^H NMR spectra of a) **1a**, b) **2**, and c) **5a**.

**Figure 3:**
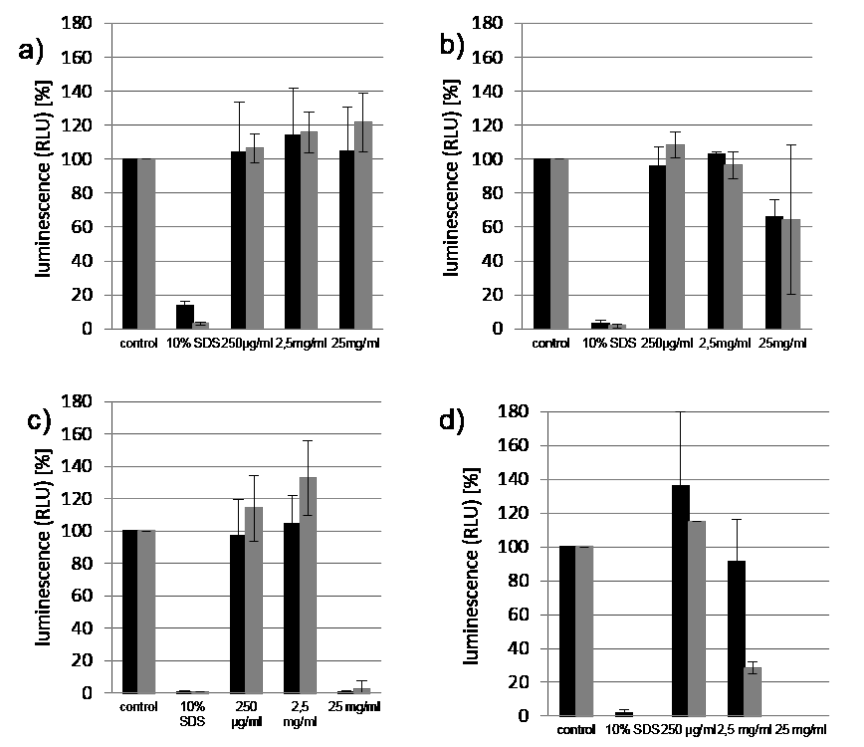
Viabilities of Caco-2 cells incubated with a) **5a** after 2 h, b) **5a** after 24 h, c) DIMEB after 2 h, d) DIMEB after 24 h, in serum (black), in basal medium (grey).

This value of DS_CD_(methyl) was confirmed by the well resolved ^1^H NMR spectrum (Figure 2). After setting the integral of the H-3 proton to 1 the signals in the range between 4.8 and 5.4 ppm integrated to a value of 12.18 protons. Taking into account the 7 anomeric protons 5.18 OH protons per CD are left, which is equivalent to a DS_CD_(methyl) = 2.2. The signals at 3.66 ppm and 3.43 ppm were assigned to the methyl groups in O-2 and O-6 position, respectively [40]. Nearly no signal was found corresponding to methyl groups in O-3 position. If the according methylation was instead performed with NaOH as the base it proceeded much faster and with a high chance of over-methylation. Careful control of the reaction time lead to another product **1b** with a similar degree of methylation DS_CD_(methyl) = 2.3, but a much higher structural heterogeneity as demonstrated by the ^1^H NMR spectrum (Figure 3s, Supporting Information File 1) which was much less resolved than the spectrum of **1a** (Figure 2a). Methylations employing NaOH as the base [41] are known to be less regioselective than those using barium hydroxide [41].

**Scheme 2:**
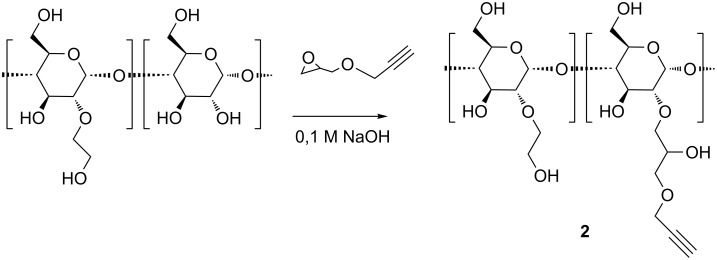
Synthesis of propargylated HES **2**.

A commercial HES with an average molar mass *M*_w_ = 130 kDa and a molar degree of substitution of MS(hydroxyethyl) = 0.4 was functionalized by reaction with propargyl glycidyl ether in water (Scheme 2) in analogy to a procedure published by Nielsen et al. for the synthesis of β-CD-dextran polymers [28]. The degree of substitution of HES with propargyl groups, DS_HES_(propg), was determined by ^1^H NMR spectroscopy from the integral of the methylene protons of the propargyl groups H-12 at 4.75 ppm relative to the ones of the anomeric proton H-01 of starch at around 5.5 ppm (Figure 2b). Surprisingly DS_HES_(propg) increased with decreasing concentration of the base NaOH. Only a moderate DS_HES_(propg) = 0.4 could be reached in 0.75 M NaOH, while a higher DS_HES_(propg) = 0.65 was accomplished in 0.1 M NaOH after 2 d reaction time. The decreasing yield was rationalized by the increasing consumption of propargyl glycidyl ether by hydrolysis with increasing OH^−^ concentration. On the other hand, due to the low p*K*_a_ ≈ 12 of polyglucanes, deprotonation still takes place at very low concentrations of OH^−^ [42–43]. Indeed the modification of HES seems to take place exclusively at the unsubstituted glucose units, because the signal of the anomeric proton H-01 of the unsubstituted glucose unit at 5.4 ppm [44] of HES (see Figure 1s, Supporting Information File 1) had nearly completely vanished in the product spectrum shown in Figure 2b. The HES derivative with DS_HES_(propg) = 0.65, **2**, was selected for the further coupling to azido-CD derivatives. Also a hydroxyethyl starch, where the CH_2_OH groups had been partially oxidized by TEMPO to carboxylate groups [45–46], was functionalized by reaction with glycidyl propargyl ether leading to a highly water soluble polymer **3** with DS_HES_(propargyl) = 0.55.

**Scheme 3:**
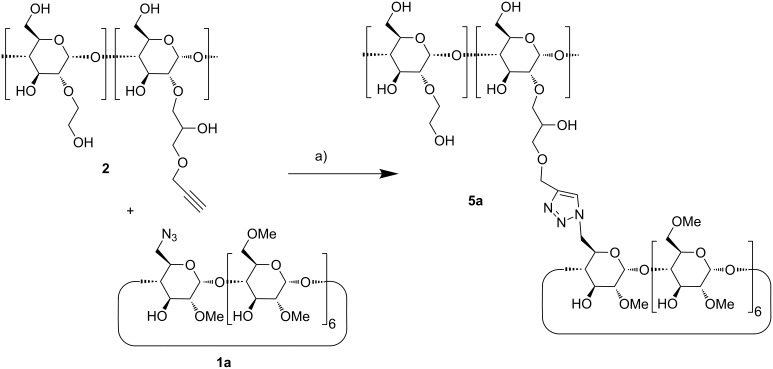
Synthesis of **5a** by [2 + 3] cycloadditon, a) CuSO_4_, ascorbate, 50 °C, 48 h.

Coupling of the CD azides **1a** and **1b** to the propargylated HES was performed by Cu^+^-catalyzed [2 + 3] cycloaddition, the so-called click reaction, leading to the corresponding triazol groups (Scheme 3). The standard protocol, introduced by Sharpless [31], using CuSO_4_ plus ascorbic acid as the catalyst already gave rise to high coupling yields. The resulting conjugates of HES and methylated CDs were isolated by ultrafiltration in nearly quantitative yields (Table 1). Nearly all propargyl groups at the HES detectable by the ^1^H NMR signal H-12 at 4.26 pm were converted to triazole groups (^1^H NMR signal H-14 at 8.03 ppm), shown in Figure 2c. The corresponding IR spectra (Figure 5s, Supporting Information File 1) revealed that the excess of azido-CD had been completely removed by the ultrafiltration step since the band of the azido group at 2102 cm^−1^ was not detectable anymore in the product. The degree of substitution of HES by CD, DS_HES_(CD), was determined from the ratio of the ^1^H NMR signals of H-14 at 8.06 ppm (0.38 protons) and of the anomeric proton H-01 of starch at 5.66 ppm.

**Table 1:** Compositions, yields, and LCST of methylated CD-HES conjugates synthesized by [2 + 3] cycloaddition.

Name	DS_CD_(Me)	DS_HES_(CD)	DS_HES_(COONa)	Yield [%]	M_CD_ [Da]	LCST [°C]

**4**	0	0.1	0	70	3410	no
**5a**	2.2	0.4	0	90	1985	52.5
**5b**	2.3	0.35	0	94	2054	84.5
**6**	2.2	0.45	0.27	95	1940	no

All of the methylated CD-HES conjugates, listed in Table 1, were clearly soluble in water at ambient conditions but most of them precipitated at elevated temperatures. This so-called lower critical solution temperature is typical for alkylated neutral polysaccharides and attributed to increasing hydrophobic interactions with increasing temperatures [47–49]. Only the CD-HES conjugate **6** did not show any precipitation below 100 °C which was attributed to the much higher hydrophilicity of the anionic carboxylate groups at the HES backbone compared to unmodified HES.

### Cytotoxicity assays

The effect of the CD polymer **5a** on the cell viability was assessed using the ATP-based CellTiter-Glo^®^ assay [50] on the human colon tumor cell line Caco-2. A first series of tests was performed in the relevant concentration range, i.e., 10× lower and up to about 10× above the clinically relevant concentration, namely 0.25 till 25 mg/mL of the polymer **5a** in the medium for 2 h and 24 h incubation times, respectively (Figure 3a,b). For comparison, the same viability test was carried out with DIMEB (Figure 3c,d).

According to DIN EN ISO 10993-5 [51], a more than 30% deviation of measurement values of treated cells compared to the untreated control was defined as cytotoxic. After treatment of the Caco-2 cell line, not any cytotoxic effect could be detected for all of the tested concentrations of **5a** after 2 h of incubation, while DIMEB was already highly toxic at 25 mg/mL. After 24 h of incubation, **5a** also showed a rather weak toxicity, while DIMEB was already highly toxic at the clinically relevant concentration (see Figure 3). The effect of the culture medium was negligible.

### Complexation of anaesthetic drugs by CD polymers

**Scheme 4:**
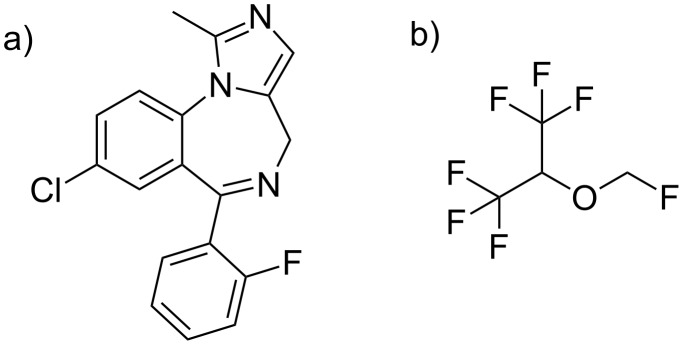
Structures of a) midazolam and b) sevoflurane.

The complexation of the anaesthetic drug sevoflurane (shown in Scheme 4) by the methylated CDs and CD polymers was quantified by measurement of the vapor pressure of the sevoflurane by gas chromatography as a function of the CD concentration as already described previously [14,52]. The respective occupancies of the hosts occupancies (the molar ratios of complexed guest vs. host) are listed in Table 2. While native β-CD shows only a weak affinity to sevoflurane, the methylated CDs RAMEB and especially DIMEB show satisfactory occupancies. Methylation increases the hydrophobicity of the CD cavity and therefore improves the compatibility with the hydrophobic guest sevoflurane. Surprisingly, the completely methylated β-CD TRIMEB had a much lower binding potential to sevoflurane than all other CD derivatives. Low binding affinities of TRIMEB are also known towards other guest molecules, such as *tert*-butyl benzoate and adamantane-1-carboxylate, and had been rationalized by the lack of intramolecular hydrogen bonds which would otherwise rigidify the CD scaffold [16]. The occupancies of the HES polymers (see Table 2) also increased with increasing degree of methylation, DS_CD_(CH_3_) similar to the monomeric CDs. The anionic polymer **6** showed a somewhat smaller binding potential.

**Table 2:** Occupancies for methylated CDs and CD polymers.

Host	DS_CD_(CH_3_)	Sevoflurane	Midazolam
		[mol %]	[mol %]

β-CD	0	32	2
RAMEB	1.8	47	6
DIMEB	2.0	69	18
TRIMEB	3.0	8	<1
**4**	0	34	<3
**5a**	2.2	67	24
**5b**	2.3	67	26
**6**	2.2	47	19

The complexation of the anaesthetic drug midazolam (shown in Scheme 4) by the methylated CDs and CD polymers was quantified by the phase solubility method [53–54]. Solubility of midazolam was measured by UV spectroscopy as the function of the concentration of the CD derivative. The resulting occupancies are listed in Table 2. The value for native β-CD was quite low and in accordance with results previously obtained by ^1^H NMR spectroscopy [55]. Again DIMEB showed the highest value. Occupancies of the polymers were in the same order of magnitude or even higher than for the respective CD monomers (Table 2).

## Conclusion

The attachment of DIMEB to a polymer backbone by the click-reaction is a very efficient way to synthesise polymeric hosts with both low toxicity and excellent binding potential. The conjugate of DIMEB and HES **5a** is regarded as a good candidate for oral or parenteral delivery of hydrophobic drugs.

## Experimental

**Complexation of sevoflurane.** The vapor pressure of sevoflurane was determined by head space gas chromatography with a Shimadzu GC-17A GC equipped with a head space unit from Shimadzu, Japan. Vials of 5 mL volume were used, the ratio between gas (*V* = 3.2 mL) and aqueous (*V* = 1.8 mL) phase was *f* = 1.77. Occupancies were calculated from the GC data as described previously [56].

**Complexation of midazolam.** An excess amount of midazolam was added to aqueous solutions containing various concentrations of CD polymers and stirred for 24 h at 25 °C. After equilibration, aliquots of the suspension were filtered through a 0.20 μm membrane cellulose filter, suitably diluted and analyzed using UV spectroscopy. The concentration of midazolam in each solution was determined by measuring its absorbance at 240 nm (ε = 21,240 M^−1^cm^−1^). The intrinsic absorption of each CD polymer was taken into account.

**Cell viability** was determined using CellTiter-Glo^®^ assay (Promega, #G7571) and Caco-2 colon carcinoma cells (DSMZ #ACC-169; passage 9-17). Cells were cultured as described by the supplier, seeded with 5 × 10^3^ cells per well in 96-well plates and tests carried out at about 80% cell confluency (~3–4 days). The assay results in cell lysis and generation of a luminescent signal proportional to the amount of ATP present, which is directly proportional to the number of cells in culture. The luminescence signal was measured in a plate reader (infinite M200, TECAN, Männedorf, Swizerland). **5a** and DIMEB were each solved in standard basal medium (MEM) or human serum and cells were treated for 2 h and 24 h with the above-mentioned concentrations. For viability assessment, the test substances were removed by washing with PBS buffer (Sigma^®^), the cells in each well were overlaid with 100 µL of basal medium and 100 μL of CellTiter-Glo^®^ reagent and luminescence was measured after 2 min of shaking and 10 min incubation at room temperature in the TECAN plate reader. All results presented are based on at least three independent biological tests. Statistical significance was determined by analysis of variance and *p* < 0.05.

**Materials:** Hydroxyethyl starch (HES) with an average molar mass (*M*_w_) of 130 kDa and a molar substitution of 0.4 was kindly provided by Fresenius Kabi, Friedberg, Germany (batch no: 17120211). β-CD was obtained from Wacker Chemie GmbH, München, Germany. HES and CD were used after drying overnight at 70 °C under reduced pressure. All other reagents and solvents were purchased from commercial suppliers and used as received. Mono-6-deoxy-6-azido-β-CD was synthesized according to a literature protocol [57]. All complexation studies were performed in saline HEPES-buffer solution (pH 7.4) with a NaCl concentration of 0.9 wt %.

**Mono-6-deoxy-6-azido-heptakis(2,6-di-*O*-methyl)-β-cyclodextrin (1a):** 3.0 g (2.59 mmol) mono-(6-deoxy-6-azido)-β-CD was dissolved in 150 mL DMF/DMSO 1:1 (v/v) under N_2_ and a mixture of 26 g (168 mmol) BaO and 26 g Ba(OH)_2_ (153 mmol) was added in small portions within 10 min under stirring. The mixture was cooled to 0 °C and 30 mL (0.32 mol) dimethyl sulfate was added over a period of 1 h under intensive stirring keeping the internal temperature below 5 °C. Stirring was continued for 4 d at rt. Then the mixture was warmed up to 85 °C for 30 min. After cooling to rt, excess dimethyl sulfate was decomposed by addition of 50 mL of 25 wt % aqueous ammonia. The mixture was stirred for 16 h and neutralized by addition of conc. HCl. Then 180 mL chloroform was added, filtered and the precipitate was washed with further 80 mL chloroform. The filtrate was concentrated to dryness in vacuum, dissolved in water, purified by nanofiltration with water against a polyethersulfone membrane (cut-off: 1 kDa) and lyophilized.

Yield: 2.5 g (1.86 mmol, 72%); DS: 2.2; ^1^H NMR (400 MHz, CDCl_3_) δ 5.25–4.94 (m, 12H, H-1/1‘, OH-3/3‘), 3.94–3.92 (m, 7H, H-3/3‘), 3.75–3.40 (m, 74H, H-4/4‘, H-5/5‘, H-6/6’a/6’b, H-7/7‘, H-8, H-9), 3.29–3.21 (m, 7H, H-2/2‘); ^13^C NMR (100 MHz, CDCl_3_) δ 101.3 (C-1), 83.5 (C-4), 82.0 (C-2), 73.2 (C-3), 70.8 (C-6a/b), 70.3 (C-5), 61.7 - 58.2 (C-7, C-8, C-9), 51.8 (C-6’a/b); MS (*m*/*z*): [M + Na^+^] 1364.63, C_55_H_95_N_3_O_34_, (*m*/*z*): [M + Na^+^ + CH_3_] 1378.02, C_56_H_97_N_3_O_34_, (*m*/*z*): [M + Na^+^ + 2CH_3_] 1392.64, C_57_H_99_N_3_O_34_, most intense peak (*m*/*z*): [M + Na^+^ + 3CH_3_] 1406.29, C_58_H_101_N_3_O_34_, (*m*/*z*): [M + Na^+^ + 4CH_3_] 1420.20, C_59_H_103_N_3_O_34_; IR 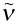 (cm^−1^): 3412 (OH), 2930 (CH), 2102 (N_3_), 1453 (CH), 1361 (OH).

**(3-Propargyloxy-2-hydroxypropyl)-hydroxyethyl starch (2):** 3.412 g (18.98 mmol) HES was dissolved in 40 mL 0.1 M NaOH and 1.8 mL (16.70 mmol) glycidyl propargyl ether were added. The temperature was increased to 35 °C and the solution was stirred. After 24 h further 1.8 mL (16.70 mmol) glycidyl propargyl ether were added and stirred for another 16 h. The product was precipitated in 500 mL 2-propanol, filtered and washed with 200 mL 2-propanol. The product was purified by ultrafiltration with water against a polyethersulfone membrane (cut-off: 5 kDa) and freeze-dried.

Yield: 3.61 g (14.97 mmol, 79%); DS: 0.65; ^1^H NMR (400 MHz, DMSO-*d*_6_/D_2_O) δ 5.68–5.38 (m, 1H, H-01/01‘), 4.27 (s, 1.30H, H-12‘), 4.03–3.45 (m, 10H, H-02/02‘, H-03/03‘, H-04/04‘, H-05/05‘, H-06/06‘, H-07/07‘, H-08/08,H-9‘, H-10‘, H-11‘, H-14‘); IR 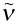 (cm^−1^): 3400.25 (OH), 2925.80 (CH), 2113.83 (-C≡C), 1456.15 (CH), 1365.50 (OH).

**Conjugate of DIMEB and HES 5a:** Under an atmosphere of N_2_ 1.6 g (1.19 mmol) **1a** was dissolved in 40 mL of degassed DMSO/H_2_O 1:1 (v/v) and 600 mg (2.4 mmol) **2** and 334 µL (119 µmol) of a solution of sodium ascorbate in water (70 mg/mL) were added. After reaching 50 °C, 211 µL (59 µmol) of a solution of CuSO_4_·5H_2_O in water (70 mg/mL) was added. The solution was stirred for 48 h and purified by ultrafiltration with water against a polyethersulfone membrane (cut-off: 5 kDa) and freeze-dried.

Yield: 1.67 g (2.15 mmol, 90%); DS: 0.4; ^1^H NMR: (400 MHz, D_2_O) δ 8.03 (s, 0.4H, H-14‘), 5.58 (m, 1H, H-01/01‘), 5.14 (m, 2.8H, H-1/1‘), 3.86–3.07 (m, 45H, H-02/02‘–H-08/08‘, H2/2‘–H-7/7‘, H8, H9‘–H12‘); IR 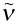 (cm^−1^): 3400.25 (OH), 2925.80 (CH), 1456.15 (CH), 1365.50 (OH).

## Supporting Information

File 1 General methods and experimental procedures for compounds **1b**, **3**, **4**, **5b** and for the oxidation of HES.

File 2 NMR spectra of HES, **1a**, **1b**, **6**, and IR spectra of HES, **2**, **5a**.
