# Supplementary material for: Conjugates of methylated cyclodextrin derivatives and hydroxyethyl starch (HES): Synthesis, cytotoxicity and inclusion of anaesthetic actives
Source: Beilstein J Org Chem. 2014 Dec 19;10:3087–96. doi: 10.3762/bjoc.10.325 (PMC4311715; doi:10.3762/bjoc.10.325)
Supplement: File 1 — General methods and experimental procedures for compounds 1b, 3, 4, 5b and for the oxidation of HES. [file Beilstein_J_Org_Chem-10-3087-s001.pdf]

Supporting Information File 1

for

Conjugates of methylated cyclodextrin derivatives and hydroxyethyl starch (HES): Synthesis, cytotoxicity and inclusion of anaesthetic actives

Lisa Markenstein[1], Antje Appelt-Menzel[2], Marco Metzger[2] and Gerhard Wenz[1*]

Address: [1]Organic Macromolecular Chemistry, Saarland University, Campus C4.2, 66123 Saarbrücken, Germany and [2]Department of Tissue Engineering and Regenerative Medicine, University Hospital Würzburg, Röntgenring 11, 97070 Würzburg, Germany

Email: Gerhard Wenz - g.wenz@mx.uni-saarland.de

*Corresponding author

General methods and experimental procedures for compounds 1b, 3, 4, 5b and for the oxidation of HES

General methods. NMR spectra were recorded in D_2O or DMSO-d_6 solution at r.t. on a BrukerBioSpin spectrometer 400 MHz Ultra shield plus (^1H: 400 MHz, ^{13}C: 100.6 MHz). The chemical shifts (δ) are given in parts per million (ppm) using the solvent peak as internal standard. Data were evaluated with ACDLabs 10.0 from Advanced

Chemistry Development Inc., Toronto, Canada. The proton and carbon atoms of the glucose units were marked with 1, 2, 3 etc. starting from the anomeric proton/carbon. The multiplicities were assigned as follows: s for singlet, d for doublet, t for triplet, bs for broad signal and m for multiplet. Mass spectra were recorded by a LC MS spectrometer ZQ-4000 from Waters GmbH, Germany, operated in ESI+ and ESI- mode. IR spectra of solid samples were obtained on a Bruker Tensor 27 Fourier-transform infrared (FT-IR) spectrometer equipped with a Golden Gate diamond attenuated total reflectance (ATR) accessory using the software OPUS by Bruker.

The purification of the polymers was performed by cross-flow ultrafiltration using Vivaflow 200 (PES) membranes from Sartorius Stedim Biotech GmbH, Germany, with a cut-off at 5 kDa. For nanofiltration of monomeric CD derivatives a NADIR® NP010 membrane from MICRODYN-NADIR GmbH, Germany, was used with a cut-off at 1kDa. Freeze-drying was carried out with a lyophilizer Lyophille Alpha 1-4 from Christ, Germany. The concentration of midazolam were determined with a UV/VIS spectrometer Evolution 220 from Thermo Scientific, USA.

Synthetic procedures

Mono-6-deoxy-6-azido-*O*-methyl-β-cyclodextrin (1b)

5.80 g (5 mmol) of mono-6-deoxy-6-azido-β-CD was dissolved under N_2 in 100 mL absolute DMF under stirring. Then 3.12 g (78 mmol) sodium hydroxide was added portionwise over 10 minutes. The solution was then cooled to -10 °C. 10.25 g (81.25 mmol) dimethyl sulfate was added dropwise and stirred for further 4 h at a temperature of -10 °C. The mixture was warmed to 85 °C for 30 min. After cooling to r.t., the excess of dimethyl sulfate was decomposed by the addition of 20 mL 25 wt.

% aqueous ammonia solution and the mixture was stirred for 16 h. The solvent was removed, the crude product was dissolved in 100 mL water and the reaction mixture was extracted three times with 150 mL of methylene chloride. The organic phase was washed once with 100 mL brine and dried over sodium sulfate. After removal of the solvent, the product was dried in vacuum.

Yield:	6.64 g (4.78 mmol, 96%)
DS	2.3
^{1}H-NMR:	(400MHz, $CDCl_3$)
	δ = 5.25 – 4.94 (m, 11H, H-1/1‘, OH-3/3‘), 3.94 – 3.92 (m, 7H, H-3/3‘), 3.75 – 3.40 (m, 68H, H-4/4‘, H-5/5‘, H-6/6’a/6’b, H-7/7‘, H-8, H-9), 3.29 – 3.21 (m, 7H, H-2/2‘).
^{13}C-NMR:	(100 MHz, $CDCl_3$)
	δ = 101.3 (C-1), 83.6 (C-4), 81.6 (C-2), 73.2 (C-3), 70.9 (C-6a/b), 70.3 (C-5), 60.4 - 59.1 (C-7, C-8, C-9), 51.3 (C-6’a/b).
MS	m/z 1364.63 (M+Na^+ [$C_{55}H_{95}N_3O_{34}$] ,
	m/z 1378.79 (M+Na^++CH_3 [$C_{56}H_{97}N_3O_{34}$] ,
	m/z 1391.83 (M+Na^+ +2CH_3[$C_{57}H_{99}N_3O_{34}$] ,
	m/z 1406.51 (M+Na^+ +3CH_3 [$C_{58}H_{101}N_3O_{34}$] , most intense peak
	m/z 1420.43 (M+Na^++4CH_3 [$C_{59}H_{103}N_3O_{34}$],
	m/z 1435.04 (M+Na^++4CH_3 [$C_{60}H_{105}N_3O_{34}$]

IR $\tilde{\nu}$ [cm^{-1}] = 3400 (OH), 2926 (CH), 2102 (N_3), 1456 (CH), 1365 (OH).

Oxidized 3-propargyl-2-hydroxypropyl-hydroxyethyl starch (3)

3.4 g (16.44 mmol) oxidized HES (DS 0.27) was dissolved in 40 mL 0.1M NaOH and 1.8 mL (16.70 mmol) glycidyl propargyl ether was added. The temperature was increased to 35 °C and the solution was stirred. After 24 h 1.8 mL (16.70 mmol) glycidyl propargyl ether was added again and stirred for 16 h. The product was precipitated in 500 mL of 2-propanol, filtered and washed with 200 mL of 2-propanol. The product was purified by ultrafiltration with water against a polyethersulfone membrane (cut-off: 5kDa) and freeze-dried.

Yield: 4.02 g (14.97 mmol, 91%)

DS: 0.55

^{1}H-NMR: (400 MHz, D_2O)

δ = 5.58 – 5.42 (m, 1H, H-01/01‘), 4.19 (s, 1.10H, H-12‘), 3.99 – 3.42 (m, 10H, H-02/02‘, H-03/03‘, H-04/04‘, H-05/05‘, H-06/06‘, H-07/07‘, H-08/08, H-9‘, H-10‘, H-11‘), 2.85 (bs, H-14‘).

IR: $\tilde{\nu}$ [cm^{-1}] = 3400 (OH), 2926 (CH), 2114 (-C≡C), 1456 (CH), 1366 (OH).

Conjugate of β-CD and HES 4

Under N_2 510 mg (0.4 mmol) mono-(6-deoxy-6-azido)-β-CD was dissolved in 40 mL of degassed DMSO/H_2O 1:1 (v/v) and 250 mg (1 mmol, DS 0.4) **2** and 500 µL (50 µmol) of a solution of sodium ascorbate in water (20 mg/mL) were added. After reaching 50 °C, 5 µL (1.4 µmol) of a solution of $CuSO_4$*5H_2O in water (70 mg/mL) was added. The solution was stirred for 48 h, purified by ultrafiltration with water against a polyethersulfone membrane (cut-off: 5kDa) and freeze-dried.

Yield: 260 mg (0.07 mmol, 70%)

DS: 0.1

^{1}H-NMR: (400 MHz, D_2O)

δ = 7.97 (s, 0.14H, H-14‘), 5.56 – 5.27 (m, 1H, H-01/01‘), 4.94 – 4.88 (m, 1H, H-1/1‘), 3.86 – 3.07 (m, 14H, H-02/02‘ – H-08/08‘, H2/2‘ – H-7/7‘, H8, H9‘ – H12‘).

IR: $\tilde{\nu}$ [cm^{-1}] = 3400 (OH), 2926 (CH), 1456 (CH), 1365 (OH).

Conjugate of RAMEB and HES 5b

Under an atmosphere of N_2 2.8 g (2.02 mmol) **1b** was dissolved in 40 mL of degassed DMSO/H_2O 1:1 (v/v) and 800 mg (3.4 mmol) **2** and 334 µL (119 µmol) of a solution of sodium ascorbate in water (70 mg/mL) were added. After reaching 50 °C, 211 µL (59 µmol) of a solution of $CuSO_4$*5H_2O in water (70 mg/mL) was added. The solution was stirred for 48 h and purified by ultrafiltration with water against a polyethersulfone membrane (cut-off: 5kDa) and freeze-dried.

Yield: 2.83 g (3.28 mmol, 96%)

DS: 0.35

^{1}H-NMR: (400 MHz, D_2O) δ = 8.03 (s, 0.4H, H-14‘), 5.58 (m, 1H, H-01/01‘), 5.14 (m, 2.8H, H-1/1‘), 3.86 – 3.07 (m, 45H, H-02/02‘ – H-08/08‘, H2/2‘ – H-7/7‘, H8, H9‘ – H12‘).

IR: $\tilde{\nu}$ [cm^{-1}] = 3400 (OH), 2925 (CH), 1456 (CH), 1365 (OH).

Conjugate of DIMEB and oxidized HES 6

Under N_2 1 g (0.72 mmol) **1a** was dissolved in 15 mL of degassed DMSO/H2O 1:1 (v/v) and 0.25 g (1.02 mmol) **3** and 138 µL (70 µmol) of a solution of sodium ascorbate in water (70 mg/mL) were added. After reaching 50 °C, 100 µL (40 µmol) of a solution of $CuSO_4$*5H_2O in water (70 mg/mL) was added. The solution was stirred for 48 h, purified by ultrafiltration with water against a polyethersulfone membrane (cut-off: 5kDa) and freeze-dried

Yield: 850 mg (0.97 mmol, 95%)

DS: 0.45

^{1}H-NMR: (400 MHz, D_2O)

δ = 8.04 (s, 0.45H, H-14‘), 5.52 (m, 1H, H-01/01‘), 5.14 (m, 2.98H, H-1/1‘), 3.86 – 3.07 (m, 60.78H, H-02/02‘ – H-08/08‘, H2/2‘ – H-7/7‘, H8, H9‘ – H12‘).

IR $\tilde{\nu}$ [cm^{-1}] = 3381 (OH), 2926 (CH), 1609 (COO^-), 1454 (CH), 1364 (OH)

Oxidation of hydroxyethyl starch (HES)

10.00 g (55.62 mmol) HES was dissolved in 200 mL water. The pH was adjusted with 1 M NaOH to 8.5 and 40 mg TEMPO (4 mg/g HES) was added to the solution. 124.33 mL (241 mmol, ρ = 1,206 g/mL, 12 wt% aqueous solution) NaOCl was added in portions of 2 mL over a period of 2 h. In the meantime the pH was kept by dropwise addition of 1M NaOH constant at 8.5. 4.21 g (11.24 mmol) sodium borohydride was added and stirred for 16 h. The product was then purified by ultrafiltration with water against a polyethersulfone membrane (cut-off: 5kDa) and freeze-dried.

Yield: 8.79g (42.50 mmol, 76 %)

DS: 0.27

^{1}H-NMR: (400 MHz, D_2O)

δ = 5.68 – 5.60 (m, 1H, H-01/01‘), 4.27 – 3.36 (m, 7H, H-02/02‘, H-03/03‘, H-04/04‘, H-05/05‘, H-06/06‘, H-07/07‘, H-08/08‘).

IR: $\tilde{\nu}$ [cm^{-1}] = 3264 (OH), 1597 (COO^-), 1412 (COO^-), 1325 (OH).
