# Supplementary material for: Conjugates of methylated cyclodextrin derivatives and hydroxyethyl starch (HES): Synthesis, cytotoxicity and inclusion of anaesthetic actives
Source: Beilstein J Org Chem. 2014 Dec 19;10:3087–96. doi: 10.3762/bjoc.10.325 (PMC4311715; doi:10.3762/bjoc.10.325)
Supplement: File 2 — NMR spectra of HES, 1a, 1b, 6, and IR spectra of HES, 2, 5a. [file Beilstein_J_Org_Chem-10-3087-s002.pdf]

Supporting Information File 2

for

Conjugates of methylated cyclodextrin derivatives and hydroxyethyl starch (HES): Synthesis, cytotoxicity and inclusion of anaesthetic actives

Lisa Markenstein[1], Antje Appelt-Menzel[2], Marco Metzger[2] and Gerhard Wenz[1*]

Address: [1]Organic Macromolecular Chemistry, Saarland University, Campus C4.2, 66123 Saarbrücken, Germany and [2]Department of Tissue Engineering and Regenerative Medicine, University Hospital Würzburg, Röntgenring 11, 97070 Würzburg, Germany

Email: Gerhard Wenz - g.wenz@mx.uni-saarland.de

*Corresponding author

NMR spectra of HES, 1a, 1b, 6, and IR spectra of HES, 2, 5a

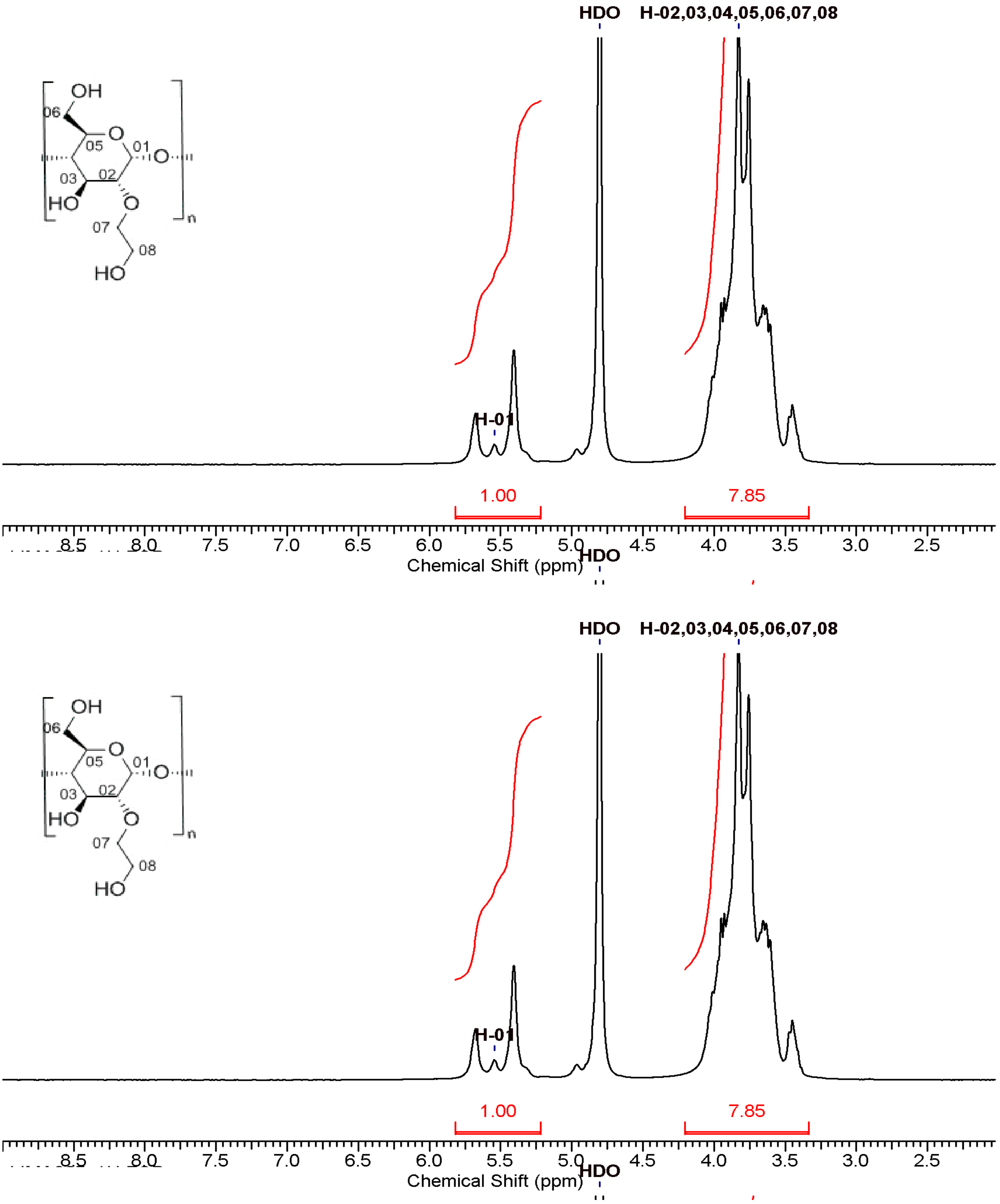

Figure 1s. ^{1}H NMR of HES in D_2O

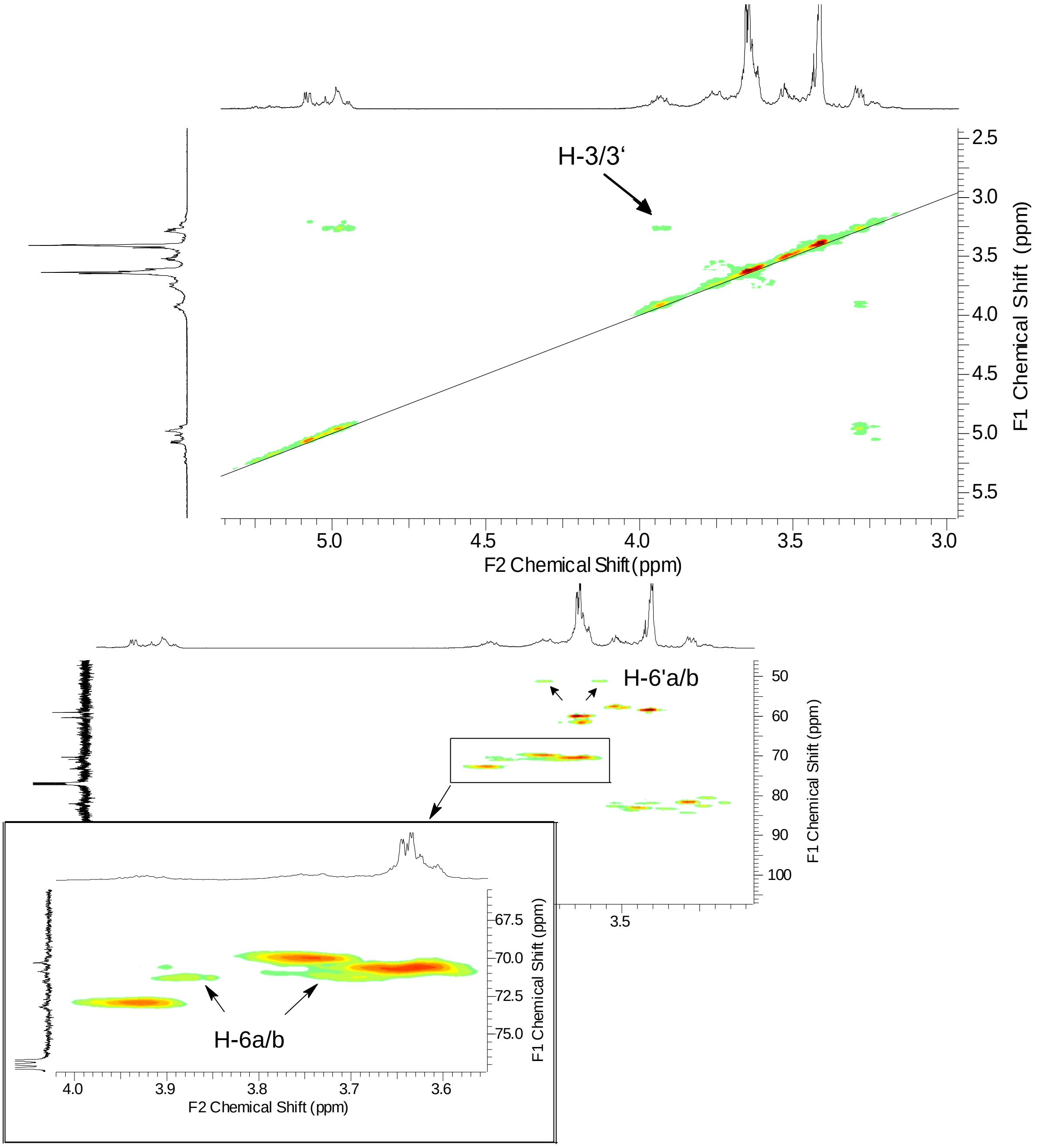

Figure 2s. ^{1}HH-COSY and HSQC spectra of azido functionalized DIMEB **1a** in D_2O

The HH-COSY indicates the distinct correlation between H-1/1’ at 5.0 ppm, H-2/2’ at 3.25 ppm and H-3/3’ at 3.92 ppm.

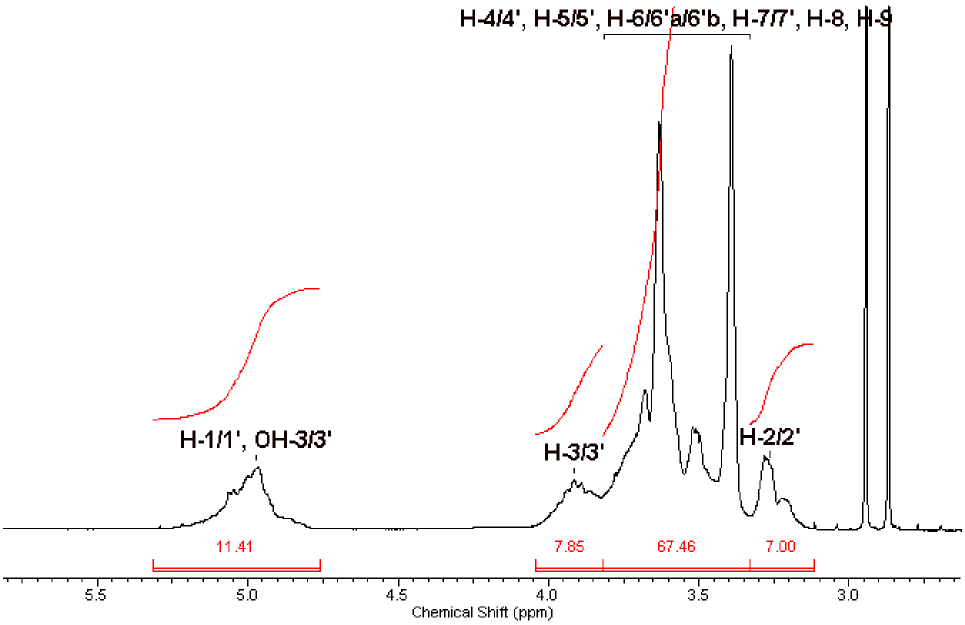

Figure 3s. ^{1}H NMR spectrum of azido functionalized RAMEB **1b** in D_2O, large signals were truncated for a better resolution of the small signals.

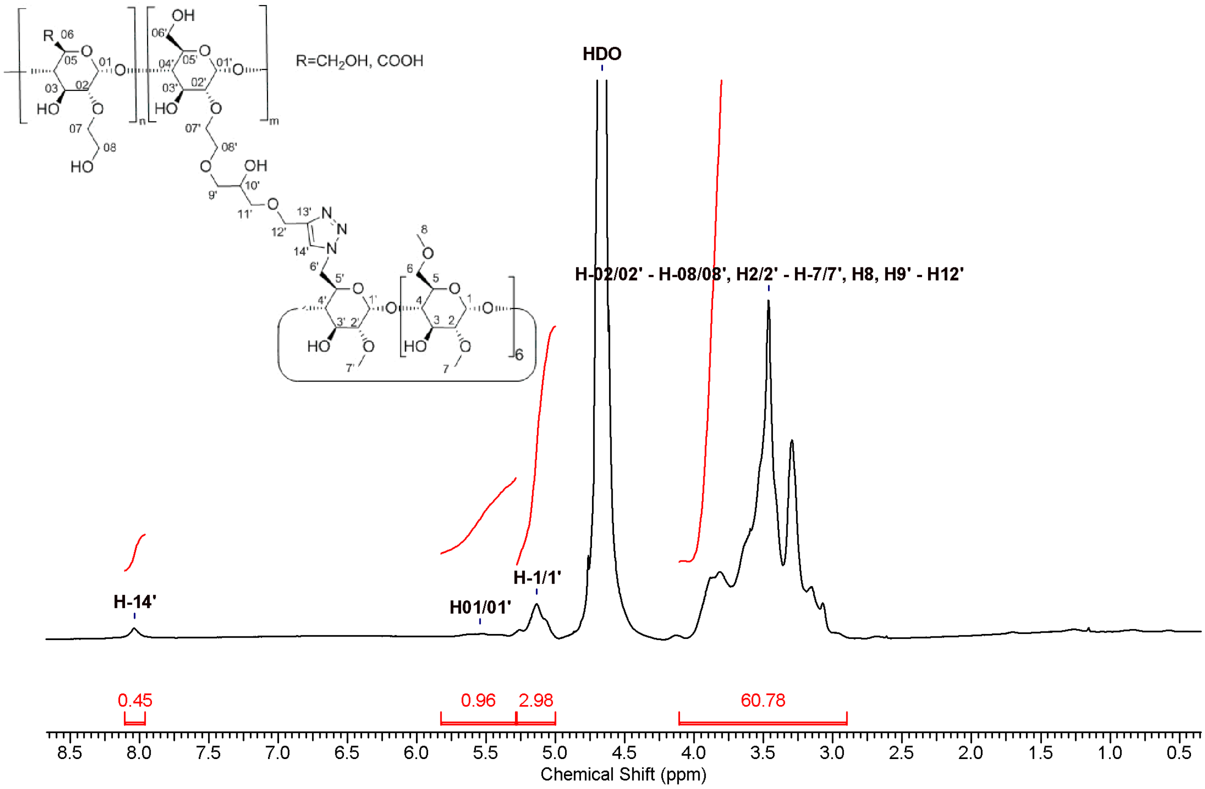

Figure 4s. ^{1}H-NMR spectrum of conjugate of DIMEB and oxidized HES **6**.

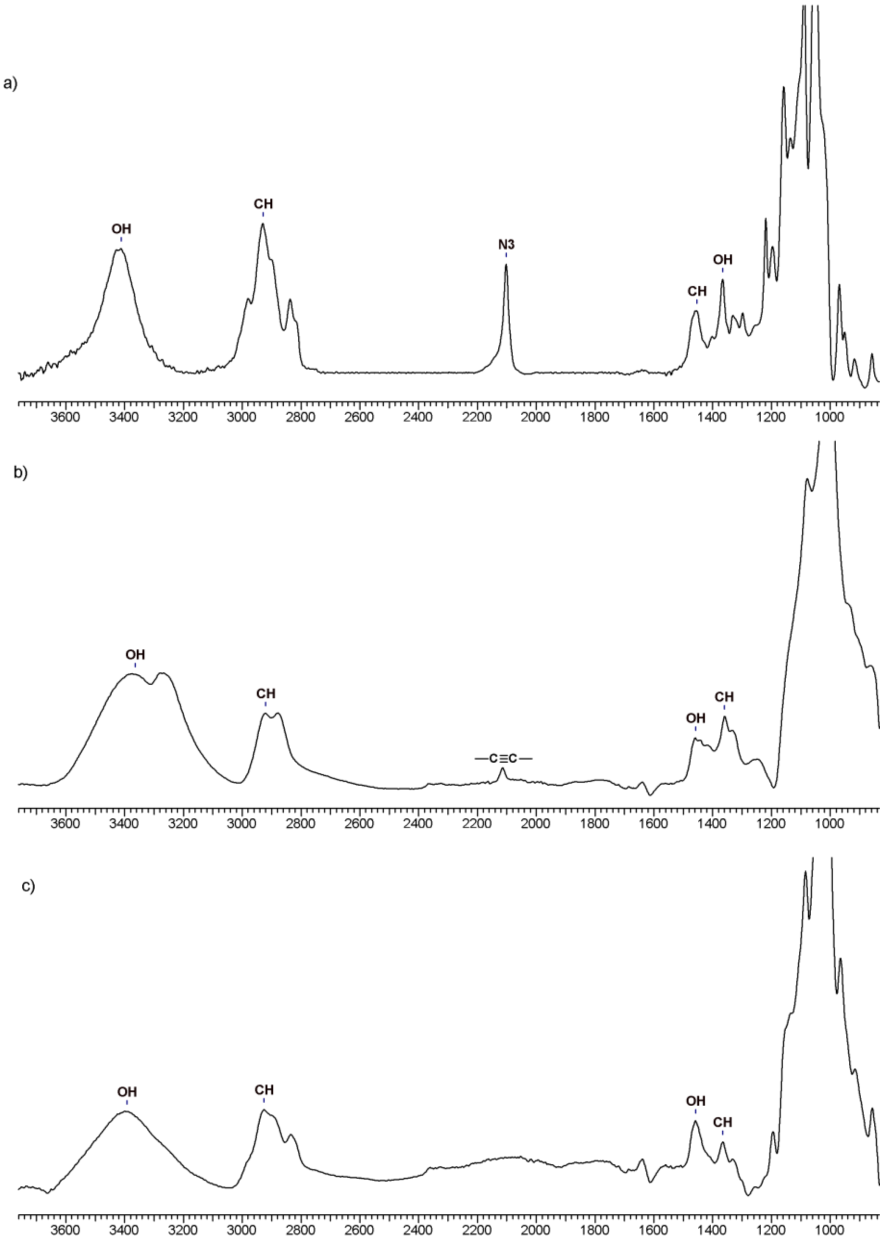

Figure 5s. FT-IR spectra of a) **1a**, b) propargylated HES **2** and c) conjugate of HES and DIMEB **5a**
